# Prevalence of Depression, Anxiety, and Stress Among Repatriated Indonesian Migrant Workers During the COVID-19 Pandemic

**DOI:** 10.3389/fpubh.2021.630295

**Published:** 2021-05-05

**Authors:** Ngakan Putu Anom Harjana, Pande Putu Januraga, Putu Ayu Indrayathi, Hailay Abrha Gesesew, Paul Russell Ward

**Affiliations:** ^1^Center for Public Health Innovation, Faculty of Medicine, Udayana University, Denpasar, Indonesia; ^2^Institute for Population and Social Research, Mahidol University, Nakhon Pathom, Thailand; ^3^Department of Public Health and Preventive Medicine, Faculty of Medicine, Udayana University, Denpasar, Indonesia; ^4^Discipline of Public Health, College of Medicine and Public Health, Flinders University, Adelaide, SA, Australia; ^5^Epidemiology, School of Health Sciences, Mekelle University, Mek'ele, Ethiopia

**Keywords:** COVID-19, mental health, migrant worker, Indonesia, prevalence

## Abstract

**Introduction:** Repatriated Indonesian migrant workers are vulnerable to developing serious mental health problems during the COVID-19 pandemic. This study aimed to assess the prevalence and associated factors of depression, anxiety, and stress among these populations during the COVID-19 pandemic.

**Methods:** Guided by the health belief model, a cross-sectional study design was employed among 335 participants, and primary data were collected through an online survey. Measured using DASS-21, anxiety, depression, and stress were the dependent variables. We performed descriptive and inferential statistical analyses—logistic regression was used to predict independently associated variables. STATA was used to execute all data analyses.

**Results:** The prevalence of depression, anxiety, and stress among repatriated Indonesian migrant workers were 10.15, 9.25, and 2.39%, respectively. The risk of anxiety and depression was low among those aged 21–30 years old, who had completed a university degree, were married, and had quarantined for 14 days. Conversely, the risk of anxiety and depression was high among those who had bad perceived health status, high perceived susceptibility, and negative stigma perception.

**Conclusion:** The prevalence of depression, anxiety, and stress among repatriated Indonesian migrant workers was relatively low compared to the general population. The risk of anxiety and depression was low among young people, educated people, and those under effective quarantine, but the risk was high among those who had negative perceptions about their health, stigma, and susceptibility to the disease.

## Introduction

The 2019 coronavirus disease (COVID-19) was first identified as unexplained cases of pneumonia in Wuhan, China. Globally, there were about 53 million confirmed cases and 1.3 million deaths attributed to COVID-19 on 13 November 2020 (1). COVID-19 has affected both social and economic situations due to public health measures (2). Moreover, the mental health problem is another substantial negative impact caused by the multifaceted impacts of the pandemic (3, 4).

It is well-known that migrant workers have negative health outcomes involving mainly mental health and physical morbidities, and workplace accidents and injuries (5). A large number of women migrant workers have reported being sexually assaulted while they worked overseas and then return to their home countries deeply traumatized (6, 7). Furthermore, the mental health problem among migrant workers occurrs due to a series of socio-environmental variables, such as loss of social status, discrimination, and separation from the family (8).

Migrant workers were more vulnerable to suffering from mental health problems due to various concerns during the COVID-19 pandemic (9). The mental health situation of migrant workers is worsening during the COVID-19 pandemic, as they already have a lower quality of life compared to the local population (5, 10). Migrant workers also encounter more barriers in accessing health services in the host country due to government-recommended self-quarantine, inadequate health insurance, and a lack of information available in their own language (5, 11). This situation makes addressing the mental health of migrant workers during the COVID-19 pandemic complex, and is therefore a subject that has been neglected (12).

Indonesia is a country with many of its citizens working overseas as migrant workers. The repatriation of Indonesian migrant workers from the worst affected countries brings a challenge for COVID-19 prevention and control to Indonesia. About 34,300 migrant workers arrived in Indonesia by June 2020 (13). At the beginning of the COVID-19 pandemic in Indonesia, most of the COVID-19 cases were imported cases, particularly from migrant workers. There were 587 confirmed cases and 10 deaths among migrant workers in Indonesia (14). A similar situation occured in Bali Province. During the early period of the COVID-19 epidemic in Bali, transmission was predominantly from imported cases including from repatriated migrant workers (15). This situation was challenging both for migrant workers and the local government.

The COVID-19 situation will create a social transformation for migrant workers, particularly in Bali. Before the pandemic, migrant workers had a high social position. Sometimes, migrant workers were considered rich, with high-class jobs and dignity (16). After COVID-19, there was a negative stigma due to increasing COVID-19 cases among those groups. As the migrant workers sometimes experience rejection from their community, the government of Bali Province urged the public to stop stigmatizing migrant workers as “COVID-19 carriers” (17, 18). This phenomenon affected the mental well-being of the migrant workers who returned to Bali. Moreover, the uncertainty about the economic situation in Bali could worsen mental health among migrant workers. In response to this, this study sought to assess the prevalence of anxiety and depression among repatriated migrant workers in Bali during the COVID-19 pandemic. The study also aimed to identify the factors associated with anxiety and depression. The result of this study might be important for the government to formulate the best approach regarding the prevention of mental health problems, particularly among migrant workers.

## Methods

### Study Design and Setting

We conducted an online cross-sectional study between 4 and 30 June 2020 using KoBo Toolbox, a free piece of software for online and offline data collection developed by Harvard Humanitarian Initiative. Indonesian migrant workers that arrived back to Bali during the COVID-19 pandemic were the targeted population. Adults aged 18 and above, and who can read and understand Bahasa Indonesian, were considered eligible, and we recruited the participants using a simplified-snowball sampling technique. We invited the participants through social media platforms (Facebook, WhatsApp, Instagram, and Twitter) because more than 60% of the Indonesian population use those platforms, including a group of migrant workers (19).

The sample was calculated using OpenEpi Version 3 (https://www.openepi.com) assuming the following parameters: the proportion of migrants with mental health problems as 30% (20), with 5% margin of error and 95% confidence interval. Finally, the minimum sample was 304.

### Study Variables, Tools, and Measurement

The dependent variables were depression, anxiety, and stress, and were measured using the depression anxiety stress scale-21 (DASS-21) (21). The DASS-21 provides a short and concise measure for a mental health problem, which has already been validated using the Indonesian language (22). It consisted of 21 items: seven items for anxiety (item no 2, 4, 7, 9, 15, 19, and 20), seven items for depression (item no 3, 5, 10, 13, 16, 17, and 21), and seven items for stress (item no 1, 6, 8, 11, 12, 14, and 18). Each item was measured on a 4 point Likert scale ranging from 0 “never” to 3 “always.” The level of depression, anxiety, and stress was calculated by multiplying the total score of each construct (consisted of seven items) by two, then re-categorized into five levels (normal to extremely severe).

The independent variables consisted of sociodemographic characteristics and constructs of the health belief model (HBM). Sociodemographic characteristics included gender, age, educational attainment, marital status, and working experience. Gender consisted of male and female; age was grouped into four categories (<20, 21–30, 31–40, and >41 years old). Educational attainment was grouped into high school graduated and university graduated; marital status consisted of single and married, and working experience was grouped into three categories (<1, 1–5, and >5 years).

The HBM constructs used in this study consisted of perceived susceptibility, perceived severity, self-efficacy, and cues to action. Items were adapted from previous studies (23–25). Each item ranges from 1 “strongly disagree” and 5 “strongly agree” and is then summed up. The total score for each construct was then rated into two categories (low and high) based on the cut-off point using mean or median according to data distribution. Items about social support, social trust, perceived stigma, and experience on the COVID-19 test and quarantine were also adopted from previous studies (26–28).

### Statistical Analysis

Data were described using descriptive statistics using proportions, mean, and percentages. This study employed a binary logistic regression model to identify the factors associated with anxiety and depression. First, bivariate logistic regression analysis was employed to find the crude associations between independent variables and dependent variables. In the second step, all variables with *p* ≤ 0.25 in the bivariate analysis were included in the multiple logistic regression. Variables with *p* ≤ 0.05 in the multiple logistic regression were considered as independent predictors for anxiety and depression. We described the strength of the measure of association using crude odds ratio (COR) in bivariate logistic regression analysis and adjusted odds ratio (aOR) in multiple logistic regression analysis. All data analyses were performed using STATA version 12.

### Ethical Approval

This study protocol was approved by the Research Ethics Commission, Faculty of Medicine, Udayana University/Sanglah General Public Hospital, Bali Province, Indonesia (No 1147/UN14.2.2.VII.14/LT/2020). Participation in this study was voluntary and anonymous. Digital informed consent was obtained before the participant completed the online form. Each participant received IDR 25,000 phone credit if they completed the questionnaires.

## Findings

### Demographic Characteristics, COVID-19 Related Experiences, and HBM Constructs

We received 347 responses during the survey period and 12 of them were excluded due to multiple responses. Table 1 presents respondents' characteristics; most of the respondents involved were male and aged 21–30 years old. More than half of the respondents had graduated from a university and were married. Most of the respondents had worked as migrant workers for more than 5 years and more than half of them had not received compensation from their company or employer when they arrived back to Indonesia due to the COVID-19 pandemic. Overall, only 2.09% of the survey participants had ever been diagnosed with COVID-19. Furthermore, most of the respondents experienced rapid-test only and only 26.57% of them experienced both rapid-test and swab-test. After they arrived in Indonesia, most of the respondents underwent a 14-day quarantine, which consisted of self-quarantine and centralized quarantine provided by the government.

**Table 1:** Sociodemographic characteristics of Indonesian migrant workers and COVID-19 related experiences (*n* = 335).

**Variable**	***n* (%)**
**Gender**	
Male	270 (80.60)
Female	65 (19.40)
**Age group (year)**	
<20	16 (4.78)
21–30	151 (45.07)
31–40	127 (37.91)
>41	41 (12.24)
**Educational attainment**	
High school graduated	81 (24.18)
University graduated	254 (75.82)
**Marital status**	
Single	151 (45.07)
Married	184 (54.93)
**Working experience (year)**	
<1	45 (13.43)
1–5	141 (42.09)
>5	149 (44.48)
**COVID-19 status**	
Negative	328 (97.91)
Positive	7 (2.09)
**Compensation from company**	
No	181 (54.03)
Yes	154 (45.97)
**COVID-19 test history**	
Never	45 (13.43)
Rapid-test only	142 (42.39)
Swab-test only	59 (17.16)
Both rapid and swab	89 (26.57)
**Doing 14-day quarantine**	
No	34 (10.15)
Yes	301 (89.85)

Meanwhile, from Table 2, we found most of the respondents believed that their health status was good. Moreover, more than half of them had low perceived severity, low perceived susceptibility, low self-efficacy, and low cues to action. On the other hand, more than half of the respondents answered low in terms of both social support and social trust. Surprisingly, more than half of them stated they experienced rejection and perceived a negative stigma.

**Table 2:** The HBM constructs and psychosocial factors of Indonesian migrant workers (*n* = 335).

**Variable**	***n* (%)**
**Perceived health status**	
Good	254 (75.82)
Bad	81 (24.18)
**Perceived severity**	
Low	207 (61.79)
High	128 (38.21)
**Perceived susceptibility**	
Low	186 (55.52)
High	149 (44.48)
**Cues to action**	
Low	188 (56.12)
High	147 (43.88)
**Response-efficacy**	
Low	199 (59.40)
High	136 (40.60)
**Perceived social support**	
Low	185 (55.22)
High	150 (44.78)
**Social trust**	
Low	180 (53.73)
High	155 (46.27)
**Have you ever experienced any rejection?**	
No	225 (67.16)
Yes	110 (32.84)
**Perceived stigma**	
Positive	156 (46.57)
Negative	179 (53.43)

### Prevalence of Depression, Anxiety, and Stress Among Indonesian Migrant Workers

This study found that 10.15% (95% CI = 6.8–13.4) of respondents had experienced depression, and 9.25% (95% CI = 6.1–12.4) of the respondents had experienced anxiety. Furthermore, only 2.39% (95% CI = 0.7–4.0) of the respondents were reported to experience stress. As the prevalence of stress was very low compared to the prevalence of depression and anxiety, we excluded the stress variable for the logistic regression analysis.

### Factors Predicting Anxiety Among Indonesian Migrant Workers

Table 3 presents the unadjusted and adjusted odds ratio of factors linked with anxiety during COVID-19. An adjusted analysis found that being 21–30 years old, a university graduate, married, and undergoing 14-day quarantine was associated with lower anxiety. Those aged 21–30 years old were less likely to get anxiety by 73% (aOR = 0.27, 95%CI: 0.03–0.91; *p* = 0.039) compared to those aged less than 20. Similarly, university graduates were 70% less likely to get anxiety (aOR = 0.3, 95%CI: 0.1–0.89; *p* = 0.030) compared to high school graduates, and married participants were also less likely to get anxiety by 86% (aOR = 0.14, 95%CI: 0.03–0.58; *p* = 0.007) compared to single participants. Those who underwent 14-day quarantine were also 84%less likely to get anxiety by (aOR = 0.16, 95%CI: 0.03–0.98; *p* = 0.047) compared to their counterparts.

**Table 3:** Unadjusted and adjusted logistic regression analysis showing factors associated with anxiety during COVID-19 (*n* = 335).

**Variable**	**Unadjusted**		**Adjusted**	
	**OR (95% CI)**	***p-*value**	**aOR (95% CI)**	***p-*value**
**Gender**				
Male (*R*)	1			
Female	0.66 (0.28–1.56)	0.347		
**Age group (year)**				
<20 (*R*)	1		1	
21–30	0.14 (0.04–0.49)	0.002	0.27 (0.03–0.91)	0.039
31–40	0.23 (0.07–0.77)	0.017	0.85 (0.13–5.37)	0.862
>41	0.31 (0.07–1.25)	0.100	3.25 (0.35–30.47)	0.303
**Educational attainment**				
High school graduated (*R*)	1		1	
University graduated	0.34 (0.16–0.73)	0.006	0.30 (0.10–0.89)	0.030
**Marital status**				
Single (*R*)	1		1	
Married	0.42 (0.19–0.90)	0.026	0.14 (0.03–0.58)	0.007
**Working experience (year)**				
<1 (*R*)	1		1	
1–5	0.41 (0.16–1.02)	0.057	0.86 (0.22–3.38)	0.829
>5	0.26 (0.10–0.69)	0.007	0.22 (0.04–1.05)	0.058
**COVID-19 status**				
Negative (*R*)	1		1	
Positive	4.12 (0.76–22.2)	0.099	4.64 (0.37–58.17)	0.234
**Perceived health status**				
Good (*R*)	1		1	
Bad	4.55 (2.13–9.73)	0.000	2.85 (1.02–7.99)	0.046
**Perceived severity**				
Low (*R*)	1			
High	1.37 (0.65–2.89)	0.404		
**Perceived susceptibility**				
Low (*R*)	1		1	
High	2.89 (1.31–6.34)	0.008	3.14 (1.14–8.57)	0.026
**Cues to action**				
Low	2.03 (0.91–4.56)	0.085	0.44 (0.08–2.32)	0.330
High (*R*)	1		1	
**Response-efficacy**				
Low	2.53 (1.06–6.05)	0.037	1.37 (0.26–7.28)	0.711
High (*R*)	1		1	
**Compensation from company**				
No	1.20 (0.57–2.53)	0.636		
Yes (*R*)	1			
**COVID-19 test history**				
Never (*R*)	1		1	
Rapid-test only	0.44 (0.18–1.09)	0.077	2.36 (0.37–15.11)	0.366
Swab-test only	0.29 (0.08–1.02)	0.053	1.66 (0.22–12.42)	0.622
Both rapid and swab	0.19 (0.05–0.65)	0.008	0.49 (0.06–3.08)	0.497
**Doing 14-day quarantine**				
No (*R*)	1		1	
Yes	0.27 (0.11–0.66)	0.004	0.16 (0.03–0.98)	0.047
**Perceived social support**				
Low	3.05 (1.27–7.28)	0.012	2.69 (0.62–11.69)	0.186
High (*R*)	1		1	
**Social trust**				
Low	2.26 (1.00–5.06)	0.048	0.83 (0.22–3.10)	0.777
High (*R*)	1		1	
**Have you ever experienced any rejection?**				
No (*R*)	1		1	
Yes	2.06 (0.98–4.34)	0.057	1.93 (0.65–5.72)	0.237
**Perceived stigma**				
Positive (*R*)	1		1	
Negative	2.73 (1.18–6.29)	0.019	6.25 (1.81–21.57)	0.004

On the other hand, having a bad perceived health status, high perceived susceptibility, and perceived negative stigma were associated with higher anxiety. Those who felt bad about their health status were about three times more likely to get anxiety (aOR = 2.85; 95% CI = 1.02–7.99; *p* = 0.046) than those who did not. Those who had high perceived susceptibility were also three times more likely to get anxiety (aOR = 3.14; 95% CI = 1.14–8.57; *p* = 0.026) compared to those who had low perceived susceptibility. In addition, those perceiving a negative stigma were six times more likely to get anxiety (aOR = 6.25; 95% CI = 1.8–21.6; *p* = 0.004) than those who did not.

### Factors Predicting Depression Among Indonesian Migrant Workers

Table 4 presents the unadjusted and adjusted odds ratio of factors linked with depression during COVID-19. An adjusted analysis found being a university graduate and doing 14-day quarantine were associated with lower depression. Those who graduated university were less likely to get depression by 77% (aOR = 0.23, 95%CI: 0.09–0.61; *p* = 0.003) compared to high school graduates. Moreover, those who underwent the 14-day quarantine were also 87%less likely to get depression (aOR = 0.13, 95%CI: 0.02–0.69; *p* = 0.017) compared to those not doing quarantine. On the other hand, those who perceived negative stigma were 11 times more likely to get depression (aOR = 10.9; 95% CI = 3.02–39.37; *p* = 0.000).

**Table 4:** Unadjusted and adjusted logistic regression analysis showing factors associated with depression during COVID-19 (*n* = 335).

**Variable**	**Unadjusted**		**Adjusted**	
	**OR (95% CI)**	***p-*value**	**aOR (95% CI)**	***p-*value**
**Gender**				
Male (*R*)	1			
Female	0.76 (0.33–1.76)	0.522		
**Age group (year)**				
<20 (*R*)	1			
21–30	0.41 (0.10–1.62)	0.203		
31–40	0.58 (0.15–2.27)	0.435		
>41	0.34 (0.06–1.91)	0.222		
**Educational attainment**				
High school graduated (*R*)	1		1	
University graduated	0.31 (0.15–0.64)	0.002	0.23 (0.09–0.61)	0.003
**Marital status**				
Single (*R*)	1		1	
Married	0.47 (0.23–0.97)	0.043	0.42 (0.16–1.15)	0.093
**Working experience (year)**				
<1 (*R*)	1		1	
1–5	0.37 (0.15–0.95)	0.039	0.52 (0.15–1.83)	0.308
>5	0.38 (0.15–0.97)	0.042	0.45 (0.12–1.78)	0.258
**COVID-19 status**				
Negative (*R*)	1		1	
Positive	3.70 (0.69–19.85)	0.127	4.05 (0.46–35.48)	0.207
**Perceived health status**				
Good (*R*)	1		1	
Bad	2.81 (1.35–5.83)	0.006	1.58 (0.62–4.03)	0.342
**Perceived severity**				
Low (*R*)	1			
High	1.31 (0.64–2.68)	0.455		
**Perceived susceptibility**				
Low (*R*)	1		1	
High	1.67 (0.82–3.40)	0.161	1.96 (0.79–4.87)	0.150
**Self-efficacy**				
Low	1.13 (0.55–2.32)	0.738		
High (*R*)	1			
**Cues to action**				
Low	2.41 (1.05–5.48)	0.037	1.70 (0.56–5.16)	0.348
High (*R*)	1		1	
**Compensation from company**				
No	1.89 (0.89–4.02)	0.097	1.67 (0.67–4.27)	0.271
Yes (*R*)	1		1	
**COVID-19 test history**				
Never (*R*)	1		1	
Rapid-test only	0.37 (0.14–0.95)	0.038	2.65 (0.48–14.53)	0.262
Swab-test only	0.45 (0.15–1.38)	0.164	2.74 (0.42–17.79)	0.291
Both rapid and swab	0.34 (0.12–0.99)	0.047	1.99 (0.34–11.78)	0.488
**Doing 14-day quarantine**				
No (*R*)	1		1	
Yes	0.31 (0.13–0.75)	0.009	0.13 (0.02–0.69)	0.017
**Perceived social support**				
Low	2.90 (1.27–6.62)	0.011	1.97 (0.50–7.80)	0.335
High (*R*)	1		1	
**Social trust**				
Low	2.23 (1.03–4.83)	0.042	1.16 (0.34–3.98)	0.814
High (*R*)	1		1	
**Have you ever experienced any rejection?**				
No (*R*)	1		1	
Yes	2.56 (1.25–5.23)	0.010	2.09 (0.82–5.38)	0.125
**Perceived stigma**				
Positive (*R*)	1		1	
Negative	7.65 (2.63–22.25)	0.000	10.90 (3.02–39.37)	0.000

## Discussion

The results of this study provide meaningful findings to help policymakers and program developers to address the main factors that might influence the mental health status of the repatriated Indonesian migrant workers. This study found that the prevalence of anxiety, depression, and stress among migrant workers was 9.25, 10.15, and 2.39%, respectively. This was relatively lower than the mental health problem among the general population during the epidemic situation, which was about 30.3% for depression, 36.1% for anxiety, and 32.1% for stress (29). Furthermore, this prevalence is also relatively low compared to healthcare workers, about 24% of whom report depression, 30% anxiety, and 40% stress (30). This is surprising as we expected higher levels of anxiety, depression, and stress due to the difficult situation experienced by the repatriated migrant workers during the COVID-19 pandemic. Reasons for the differences could come from differences in the instrument used in the studies, socio-demographic characteristics, and recruitment strategy.

The present study found that the younger age group was associated with higher anxiety, similar to the findings in Austria among the general population during the COVID-19 lockdown (31). Additionally, the current study also found that higher educational attainment was linked with lower anxiety and depression. Different levels of knowledge and experience on coping strategies might explain why the older age group and those with a higher educational attainment have lower anxiety and depression (32). Furthermore, this is because the younger age group experiences a range of changes in transition in behavior, education, and social and developmental challenges. The addition of mental health-related COVID-19 escalates mental illness among these age groups. Married people have lower anxiety compared to unmarried in the present study, which might be related to better social support from their partner or family (33, 34).

A study in China during the early point of the pandemic found different effects of quarantine on mental health status. An initial report revealed that quarantine was not related to mental health problems (35), as opposed to other studies which reported the risk of mental health problems (36). This study indicated that undergoin a 14-day quarantine was associated with lower anxiety and depression. These results might be related to the different sociodemographic situations, although is open to research to confirm the conflicting findings. According to the quarantine policy in Bali, the 14-day quarantine was recommended in the form of self-quarantine or centralized quarantine. As 64% of respondents experienced the centralized quarantine, lower anxiety and depression among migrant worker might be related to better health services provided by the government during quarantine.

There was a significant association between perceived negative stigma and higher anxiety and depression in the current study. During the COVID-19 pandemic, the negative stigma from society was commonly found among health workers due to the higher risk of disease transmission among this group (37). In the context of the repatriated migrant worker in Bali, negative stigma might have occurred due to the higher number of imported cases and local transmission among these groups during the early COVID-19 situation (17, 18). Moreover, lack of knowledge and awareness related to COVID-19 in society could increase the negative stigma related to COVID-19 (38). The resistance and misconceptions to the illness and its public health measures could also be additional arguments (39).

This study found that bad perceived health status and high perceived susceptibility were linked with anxiety. This might be because the respondent who had poor health status perceptions and higher perceived susceptibility tended to suffer from a mental health problem, compared to the respondent who perceived their health status as good and had lower perceived susceptibility (40, 41). In addition, even though this study found that perceived social support was not significantly associated with a mental health problem, other studies found that the support from family members, friends, and communities was essential to reduce mental health problems during COVID-19 pandemic (42). The level of support given and the socio-economic situation during the COVID-19 pandemic also matters substantially. This implies the need to improve on health services during quarantine and that health promotion should be implemented to reduce the negative stigma related to COVID-19. Emphasizing the needs of migrants to stay away from their family, ensuring mental and physical support, and providing basic needs and effective counseling were considered as comprehensive health services to reduce mental health problems (43). In terms of health promotion, the government should give clear, concise, and necessary information in a respectful way to reduce social stigma, including avoiding the negative impact of social media exposure related to COVID-19.

This study has the following limitations. As this study uses a cross-sectional approach with convenience sampling, it cannot imply causality and inference. The online survey which was based on self-report may be subjected to recall bias, self-bias, and a tendency to report socially desirable responses. Despite these limitations, this study contributes to the understanding of anxiety and depression among Balinese migrant workers. Lastly, further studies should consider exploring more about the mental health problems among migrant worker in more in-depth qualitative research. The results of this online survey could be used as baseline information to conduct qualitative studies in the future.

## Conclusions

The prevalence of depression, anxiety, and stress among repatriated Indonesian migrant workers were relatively low compared to mental health problems among the general population and healthcare workers. However, the focus should be given to particular groups of migrants including those who had bad perceived health status, high perceived susceptibility, and perceived negative stigma. Improving the health service during the quarantine and promoting general health was essential to reducign the mental health problems among migrant workers during the COVID-19 pandemic.

## Data Availability Statement

All data relevant to the study are included in the article or uploaded as Supplementary Information—No additional data is available. As part of ethical clearance, the raw data used to support the findings of this study are restricted by the Faculty of Medicine, Udayana University Review Board to protect research subject privacy.

## Ethics Statement

The studies involving human participants were reviewed and approved by Research Ethics Commission, Faculty of Medicine, Udayana University/Sanglah General Public Hospital, Bali Province, Indonesia. The patients/participants provided their written informed consent to participate in this study.

## Author Contributions

PJ conceived the idea. NH and PJ analyzed the data. NH drafted the manuscript. NH, PJ, PI, HG, and PW critically reviewed and approved the final version of the manuscript. All authors contributed to the article and approved the submitted version.

## Conflict of Interest

The authors declare that the research was conducted in the absence of any commercial or financial relationships that could be construed as a potential conflict of interest.
